# Red Tomato Products as an Alternative to Reduce Synthetic Dyes in the Food Industry: A Review

**DOI:** 10.3390/molecules26237125

**Published:** 2021-11-25

**Authors:** Tiago Alves Castro, Bruna Santos Leite, Larissa Santos Assunção, Tayane de Jesus Freitas, Nelson Barros Colauto, Giani Andrea Linde, Deborah Murowaniecki Otero, Bruna Aparecida Souza Machado, Camila Duarte Ferreira Ribeiro

**Affiliations:** 1 Department of Food Science, Nutrition School, Federal University of Bahia, Salvador 40110-907, Brazil; tiago_alves1997@hotmail.com (T.A.C.); bruna.nice@hotmail.com (B.S.L.); gianilindecolauto@gmail.com (G.A.L.); deborah.otero@ufba.br (D.M.O.); 2 Faculty of Pharmacy, Federal University of Bahia, Salvador 40170-290, Brazil; larissa.sanut@yahoo.com.br (L.S.A.); tay.jfreitas@gmail.com (T.d.J.F.); nelsonbcolauto@gmail.com (N.B.C.); 3 Laboratory of Pharmaceutical’s, National Service of Industrial Learning, University Center SENAI CIMATEC, Salvador 41650-010, Brazil; brunam@fieb.org.br

**Keywords:** natural food additive, antioxidant activity, lycopene, food pigment, carotenes

## Abstract

Most dyes used in the food industry are synthetic and can be a health hazard. Red tomato may serve as a natural alternative dye to replace synthetic colorants. This study aimed to review the literature on the addition of red tomato products (powder tomato, paste, freeze-dried, tomato peel powder, tomato pomace) to reduce the usage of synthetic dyes in the food industry. Red tomato products have been used as coloring in pasta, bologna, sausages, cookies, crackers, macaroons, hamburgers, breads, muffins, cheeses, and nuggets. The *trans-cis* isomerization of lycopene by oxidative processes directly affects the color of the pigment. The lycopene contained in tomato has antioxidant activity and could reduce or eliminate other oxidants and/or synthetic preservatives in food. Moreover, tomatoes in foods have high sensory scores, nutritional appeal, and marketing potential. However, its use as a food colorant has been not extensively explored. Therefore, further studies are still required, especially on the stability of carotenoids in tomatoes used in processed foods.

## 1. Introduction

Most of the pigments used in the food industry are synthetic. Brazil has the largest number of authorized synthetic dyes. However, there is a worldwide trend shift toward the reduction of synthetic dyes due to the search for healthier foods [1]. Moreover, some of these synthetic dyes are carcinogenic, allergenic, and mutagenic and may generate behavioral changes, such as hyperactivity, which has increased the concern of health authorities worldwide [2]. Although natural dyes are not available with the same technological and economic advantages of synthetic dyes, there is a high preference for pigments obtained from natural sources, given their nutritional values and health benefits [3].

The wastage in world food production has reached 30%, which is equivalent to approximately 1.3 billion tons of food [4]. In Brazil, 54% of tomatoes produced are lost [5] due to inadequate storage and handling from producer to consumer [4]. Millions of tons of tomatoes are processed annually to produce tomato juices, sauces, purees, pastes, and preserves, which have resulted in large amounts of industrial wastes, such as tomato peels, pulp, and seeds [6]. These by-products of vegetable food processing pose a disposal problem for the food industry; however, they can be promising sources of compounds for the food, pharmaceutical, and cosmetic industries, given their nutritional properties and potential use as colorants [7]. Therefore, tomato by-products could be used for pigment extraction and become a sustainable alternative source of natural pigments. García et al. [8] proposed the direct addition of dry tomato peel to raw and cooked hamburgers.

Tomato is rich in lycopene, which is the carotenoid responsible for the red color. Lycopene is a natural pigment with antioxidant potential and, in some cases, pro-vitamin A activity, among other biological, therapeutic, and preventive activities against several disorders and diseases in humans [9]. Therefore, the use of tomato as a dye would not only serve the purpose of food coloring but would provide functional benefits due to their antioxidant compounds, such as vitamins C and E, carotenoids, and polyphenols, which scavenge free radicals, thereby contributing to health protection and sustenance [10]. In addition, tomatoes contain trace elements, such as selenium, copper, manganese, and zinc, which act as cofactors of antioxidant enzymes [11]. Tomatoes also contain α-, β-, γ-, δ-carotenes, and other carotenoids, such as lutein, neurosporene, phytoene, and phytofluene [12], which act synergistically with lycopene [13].

Considering that synthetic pigments can cause adverse health reactions and that tomatoes or their by-products are healthy sources of bioactive compounds, they could be used in the production of red dyes for the food industry. Therefore, this study aimed to review the addition of red tomatoes in foods as strategy for reducing the usage of synthetic dyes in food industries as well as prolonging the shelf life of foods.

## 2. Why Use Tomatoes as Food Coloring?

### 2.1. Tomato

*Solanum lycopersicum* L. is the plant that produces tomato, an edible fruit belonging to the Solanaceae family, and it is native to South America but cultivated almost worldwide [14]. It has several synonyms and combinations, such as *Lycopersicon lycopersicon* (L.) H. Karst., *Amatula rubra* Medik., *Lycopersicon lycopersicum* var. *cerasiforme* (Alef.) M. R. Almeida, *Lycopersicon solanum-lycopersicum* Hill, *Amatula flava* Medik., *Solanum pseudolycopersicum* Jacq., *Lycopersicon galeni* Mill., *Solanum spurium* J. F. Gmel., *Lycopersicon humboldtii* Dunal, *Lycopersicon esculentum* subsp. *Galenii* (Mill.) Luckwill, *Lycopersicon esculentum* subsp. *Humboldtii* (Dunal) Luckwill, *Lycopersicon pyriforme* Dunal, *Lycopersicon esculentum* Mill., *Solanum pomiferum* Cav., *Solanum pyriforme* Poir., *Lycopersicon pomum-amoris* Moench, *Lycopersicon solanum* Medik., *Solanum humboldtii* Willd., *Solanum spurium* Balb., *Lycopersicon cerasiforme* Dunal, *Solanum lycopersicum* var. *cerasiforme* (Dunal) D. M. Spooner, G. J. Anderson & R. K. Jansen, *Lycopersicon esculentum* var. cerasiforme (Dunal) A. Gray, *Lycopersicon esculentum* var. *leptophyllum* (Dunal) D’Arcy, *Scubulon humboldti* Raf., and *Solanum luridum* Salisb [15].

**Figure 1:**
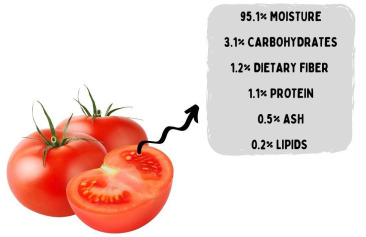
Chemical composition of tomatoes.

Brazil is among the 10 largest tomato producers in the world, with a production of 4.1 million tons in 2018 [16]. On average, tomatoes are composed of macronutrients and micronutrients (Figure 1), with an emphasis on insoluble (cellulose) and soluble (pectin) fibers, as well as organic acids (citric, malic, and ascorbic), sugars, B vitamins, minerals (Ca, K, Mg, and P), and carotenoids [17].

Chlorophylls and carotenoids are responsible for the color of tomatoes. In the early stages, chlorophylls provide a green color, which is degraded as they mature, so the color of the carotenoids stands out. Carotenoids correspond to 6–9 mg/kg of the composition of fresh tomatoes and lycopene is the main carotenoid, representing 30–200 mg/kg of fresh tomatoes. This pigment is responsible for the red color, and β-carotene, which represents about 5.44 mg/kg of fresh tomatoes, is responsible for the yellow color [18]. The lycopene content increases as the tomatoes ripen, transforming chloroplasts into chromoplasts and, as this synthesis increases, the fruit turns an intense red color [19].

The carotenoid content in tomatoes of the most consumed variety is composed of 3.2 µg/g β-carotene, 35 µg/g lycopene, and 1 µg/g lutein [20]. Furthermore, the antioxidant activity of a whole tomato is about 5.94% and that of the pulp is 8.65% [21]. In addition to carotenoids, several functional properties in tomatoes have been reported to reduce the risk of certain cancers, such as the antioxidants ascorbic acid and phenolics, which play a preventive role, especially against chronic non-transmissible diseases [22]. Tomato color parameters are usually measured by the CIELab scale according to L* (brightness), a* (redness-greenness), and b* (yellowness-blueness). The L* values are usually in the range of 40.07–42.74, a* is in the range of 17.95–29.68, and b* is the range of 27.01–29.41, which indicates the reddening degree of a ripe tomato [23].

### 2.2. Carotenoids

Carotenoids comprise a class of natural pigments with a basic structure of 40 carbon atoms in a polyene chain. In human nutrition, these compounds act as the main sources of retinol, pro-vitamin A, and antioxidant agents [24]. Carotenoids considered pro-vitamins are those with pro-vitamin A activity, while the inactive carotenoids are those with antioxidant and coloring activities [25].

The colors of these pigments range from yellow to red and are found in flowers, leaves, fruits, and some roots [26]. Some carotenoids are considered colorless or ultraviolet, since they absorb light at frequencies ranging from 350 to 400 nm [27]. To be visible to the human eye, carotenoids require at least seven conjugated bonds and the more bonds there are, the redder and more visible the color becomes [28].

Carotenoids are soluble in lipids and highly bioavailable if consumed with fatty or oily foods [29]. However, the major challenge for the industrial use of carotenoids is the isomerization process. The *trans* form has a stronger coloration, whereas the *cis* form causes color loss, which is a counterpoint in the development of dyes from carotenoids. In addition, the conversion process from *trans* to *cis* molecules are intrinsic to industrial processing, such as exposure to oxygen, heat, acids, light, and lipoxygenase enzymes [27].

#### Lycopene

**Figure 2:**
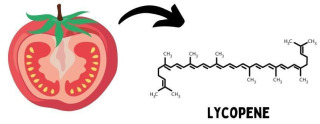
Lycopene found in tomatoes.

Lycopene is a carotenoid belonging to the non-oxygenated subgroup and is characterized by an acyclic and symmetric structure containing eleven linearly arranged conjugated double bonds. It also has two unconjugated double bonds, which gives it a greater antioxidant activity; therefore, it is considered one of the most potent biological free radical scavengers. It is found in red colored vegetables, such as tomatoes (Figure 2), watermelons, papayas, guavas, and red bell peppers, among others [30].

### 2.3. Dyes

The initial dyeing process was based on natural sources, such as leaves, flowers, mollusk secretion, animal blood, and root extracts, among others [31]. Synthetic dyes were developed in 1771 by P. Woulfe by treating indigo, a natural dye extracted from leaves of the genus *Indigofera*, with nitric acid to obtain picric acid. However, in 1858, Peter Griess created the family of azo dyes, which is a milestone in the history of synthetic dyes, and after three years, he began the large-scale process of dye production with a range of synthetic colors in the world [32].

The food industry uses food colorants in order to meet the expectations of consumers who commonly associate color to product quality [33]. The production of food colorants is growing at about 10% per year, with an estimated production of about 8 million tons per year. The food industry uses more than 2000 different types of colorants in their products and China, India, United Kingdom, and South Korea are the largest supplier of food colorants. In addition, the European continent has been investing in this market share, especially in natural colorants, due to the growing demand for clean label foods and health awareness [34,35]. Moreover, the use of colorant in food products is also associated with the ability to control the colors of products after processing and during storage time, giving them an identity color that is important to consumer preference [36].

According to the Health Surveillance Secretariat (SVS), food dyes are considered additives: that is, any ingredient intentionally added to foods for the purpose of adding physical, chemical, biological, or sensory characteristics in all stages of production until storage. Thus, food dyes are defined as substances that confer, intensify, or restore color to foods [37].

Natural sources of food dyes are vegetables, animals, and microorganisms. Synthetic dyes obtained by organic synthesis are produced after technological processes and are not found in natural sources. Synthetic dyes identical to natural dyes have a chemical structure similar to the isolated principle found in the natural source. Inorganic colorants or pigments from mineral substances are subjected to the preparation and purification processes for use in food. Caramel colorants are obtained from the controlled heating of inverted sugars or other carbohydrates in the presence of ammonia compounds and sulfites [38].

#### 2.3.1. Synthetic Dyes

Synthetic dyes are progressively replacing natural dyes due to their high stability (light, oxygen, heat, and pH), uniformity, coloring power, freedom from microbiological contamination, and relatively low production cost, apart from ensuring the uniformity of food produced on a large scale. However, the use of synthetics is now gradually declining due to its potential harm to health [39]. The controversy regarding the use of additives in foods stands between technological need and safety as well as the benefits versus potential toxicological risks of chronic exposure to these substances [2].

It has been reported that the main factor for reducing the use of synthetic additives is the increase in consumer knowledge about what they eat [40]. Furthermore, studies since 1960 have reported the toxicity of food colorings, which has raised a serious concern for world health authorities (Table 1).

The first set of global food standards and guidelines is the Codex Alimentarius (CA), which was established in early 1962 by Food and Agriculture Organization (FAO) and World Health Organization (WHO). The CA operates the Codex Committee on Food Additives (CCFA), which focuses specifically on food additives. Some of these specific functions include the preparation of risk assessment priority lists by Joint Expert Committee on Food Additives (JECFA), assigning functional classes, establishing or endorsing maximum permitted levels, considering methods of analysis for determination in food, recommending identity and purity specifications for adoption by the committee, and considering and developing norms or codes for related matters, such as labeling [51].

In 1989, the CCFA created the International Numbering System for the purpose of providing an international numerical system for identifying additives in the list of food ingredients and as an alternative to using the specific name, which is often long and complex [52].

The CA and WHO/FAO have developed a database that gathers available evidence on the biological activity of food additives, which is called General Standards for Food Additives (GSFA). The purpose of these standards is to harmonize international rules, which are useful in the context of global food marketing [51].

The main global regulators of food additives are the European Food Safety (EFSA) of the European Union (EU) and the US Food and Drug Administration (FDA) [52]. In the US, the FDA has the primary function of evaluating scientific data and information to ensure that a dye is safe for a particular purpose. Any food that contains an unapproved substance is considered adulterated by law and is subject to coercive measures to eliminate it from trade [53].

In addition, according to EU legislation, all additives must be evaluated by EFSA, an agency funded by the EU, which operates independently of legislative and executive institutions (Commission, Council, and Parliament) and EU member states. EFSA, as a risk assessor, produces scientific recommendations and guidelines that form the basis for European policies and legislation. Based on scientific recommendations, the Commission and European States decide whether a substance is authorized or not. When permitted, a substance becomes part of the EU list of authorized food additives [54].

In turn, in New Zealand, the safety assessment is done by Food Standards Australia New Zealand (FSANZ), which is an agency that periodically investigates the safety of food additives at the proposed levels of use and whether there is a good technological reason for its usage [55].

In Brazil, synthetic dyes are regulated by the National Health Surveillance Agency (ANVISA). Based on risk analysis principles, ANVISA establishes the additives that are allowed for different categories of food, providing their respective functions and maximum usage limits in order to ensure a desirable technological effect, without posing any considerable risk to human health. Thus, the regulatory process includes a case-by-case assessment of these substances at the request of the interested party who must present, among other information, the proof of safety of use, technological need, proposed limit, estimated intake of the additive, and internationally recognized references [56].

Although food additives are assessed based on a safety and technological effectiveness prior to their authorization for use, the globally accepted and used approach for safety assessment has several limitations, such as difficulty in translating toxicological data obtained in animal studies for humans and difficulty of predicting inter-individual variability. In addition, recent studies have suggested that these substances may cause adverse reactions that are not identified in the safety assessment, including allergic reactions, food intolerances, and hyperactivity [57,58,59]. However, some studies on the toxicity of synthetic dyes to health are still inconclusive, and the studies that bring greater scientific evidence are those conducted with animals [40].

In Brazil, in 2006, there was an alignment with MERCOSUL (Southern Common Market) through resolution GMC n. 11/2006, in which 16 dyes, such as amaranth, erythrosine, red 40, red 2G, ponceau 4R, twilight yellow FCF, tartrazine yellow, quinoline yellow, indigotin, bright blue FCF, azorubine, fast green FCF, patent blue V, shiny black BN, brown HT, and litol rubina BK, were allowed [60]. Each of these dyes has its maximum allowed limit according to its application [61]. In spite of this, Brazilian legislation is the most permissive regarding the use of food colors compared to other countries.

Studies have reported some side effects caused by the consumption of synthetic dyes, such as twilight yellow dye, which was banned in Finland and Norway for causing hyperactivity in children when associated with the preservative sodium benzoate. Bright blue was banned in Germany, Austria, France, Belgium, Norway, Sweden, and Switzerland for causing skin irritations and bronchial constriction in association with other dyes. Bordeaux red (a mixture of amaranth and bright blue) was banned in the United States, Austria, Norway, and Russia for causing asthma attacks and eczema. In some countries, red 40, ponceau red, and erythrosine red were excluded due to their association with kidney disease and anemia [62]. In 2007, in the US, red 2G and brown FK were reassessed and removed from the approved list of dyes due to the association of red 2G with carcinogenicity and the lack of safety data of brown FK [62]. According to the EU legislation, in 2009, the dusk yellow dyes FCF, ponceau 4R, and quinoline yellow had their daily rates reduced due to their toxicity in rats [63,64,65].

In Brazil, there are no documents prohibiting the use of certain synthetic dyes, such as sunset yellow (INS110—International Numbering System). This coloring agent can be used in various products, such as flavored yogurts, fillings for bakery products and cookies, confectionery products, desserts, edible ice cream, candies, confectionery, bonbons, chocolates, and similar ready-to-consume confectionery dips. In these foods, the maximum allowed limit (g/100 g or g/100 mL) according to legislation is 0.01 g/100 g [66]. According to the basic principle of good manufacturing practices, the amount of additive added to foods should be limited to the minimum amount necessary to achieve the desired technological effect. Brilliant blue dye is also used and allowed in food product categories. Some of them are ready-to-eat soups and broths as well as dehydrated soups and broths; the maximum permitted limit for these products is 0.005 g/100 [67]. However, according to Table 2, there are resolutions that prohibit or restrict the use of dyes for some types of foods and guide the maximum amount allowed [68]. The United States, which had more than 700 substances with coloring power at the beginning of the 20th century, currently allows only nine types of synthetic dyes in foods, two of which are restricted. In Japan, under current legislation, the use of eleven synthetic dyes was allowed. In Brazil, only eleven artificial colors were allowed for food and beverages [69].

Currently, authorizations for the use of food additives have been done according to the type of product. In turn, the legislation is segmented into numerous technical regulations, which makes consultation by the ANVISA, the productive sector, and consumers difficult. In addition, it can generate frequent questions to ANVISA about substances that are authorized or not. To facilitate access to legislation, the regulatory agency consolidated authorizations for both product categories and food additives. Therefore, foods were organized into 24 categories, receiving the name Brazilian System of Food Categorization. The harmonization of some technical regulations on food additives within MERCOSUL until 2008 was considered [60,70].

Food additive legislation, such as that of the US and EU, has reduced the number of synthetic dyes allowed in their regulations. However, Brazilian legislation is more permissive, and the list of authorized dyes has been increasing consistently. This may be related to the MERCOSUL agreements to facilitate the commercialization of products among member countries. With the increasing trade in food products among countries, the development of more reliable and rapid methods of analysis is required. For artificial colorings, proving that the product is artificially colored is not enough, since each colorant or their mixture must be detected and quantified individually; however, this detection and quantification is difficult due to lack of analytical methodologies. Furthermore, in the EU, dyes permitted by law are subject to reassessments in a more common way based on scientific findings. However, in Brazil, these revaluations take longer periods to happen [79].

Consumer interest in sustainable food value chains based on ethical and environmental standards, in addition to food safety, quality, and zero harmful effects on health, is increasing. Due to the emergence of this consciousness among consumers, companies are urged to integrate the aspects of sustainability in their processes [80,81,82]. Apart from producing high-quality food commodities, food businesses need to consider the environmental and social impact of their activities along the entire product life cycle and develop their strategy toward ensuring more sustainability [81,83,84]. Therefore, the life cycle of a final food product emphasizes the production of bulk ingredients and minor ingredients, such as food colorants.

In addition to health problems, the environmental issues involved are also notorious, since it has been estimated that around 15% of the world’s dye production is lost to the environment during the synthesis, processing, or application of these dyes [39]. Umbuzeiro et al. [85] reported several mutagenic activities due to the effluents from a dye processing industry that is near a river in São Paulo, Brazil, such that, without proper treatment, it will pose an environmental and human health risk [39]. Moreover, most synthetic dyes are not eliminated by conventional water treatments, such as pre-chlorination, flocculation, coagulation, flotation, decantation, and filtration. Synthetic dyes can be found in drinking water at a range of 1.65–316 ng/L, with potential mutagenic activity [86]. Since water bodies receive significant amounts of effluents, it is important to develop alternatives to remove compounds that are not easily eliminated by conventional water treatment processes. The contamination of rivers is a result of inefficient legislation and/or inspections [39].

#### 2.3.2. Natural Dyes

The food industry is in search for natural pigments that can be used as alternatives to synthetic dyes. This trend has been observed since the 1990s, as 54.2% of Brazilian dye industries were already engaged in the production of natural dyes. The most used are annatto, turmeric, carmine, beet red, paprika, anthocyanin, and chlorophyll [1]. The advantage of using principles extracted from vegetables goes beyond its coloring effect, since it can also bring functional effects and preservation qualities to the processed food [87]. In addition to their technological advantages, natural dyes are more sustainable, since they can be found in edible by-products or residues, such as vegetable peels and vegetables without commercial value, mainly in the family farming system of production [39]. Studies on the effectiveness of replacing synthetic dyes with natural ones are urgently required, considering that synthetic dyes are non-renewable, non-biodegradable, and can cause toxic waste pollution, thereby posing a huge challenge in disposal of by-product wastes in a cost-effective manner [88,89]. The sustainability of food colorants can be evaluated by some indicators, such as the procurement of raw material, material exploitation, energy, water consumption during production, recovery of by-products, recirculation, and naturalness [90].

According to the magazine *Additives & Ingredients* [91], the most frequently used and consumed synthetic dyes are from the family of azo compounds, which correspond to the color variation from yellow to red. As a natural alternative, these colors can be found in abundance from substances in the carotenoid family. It is estimated that nature produces about 100 million tons of carotenoids annually [1]. However, it is still a challenge for the food industry to develop natural pigments with stability to light, oxygen, heat, and pH. Therefore, some studies that deal with technological solutions to provide stability to natural dyes, such as the encapsulated natural dyes that favor uniformity, standardization, and solubility in water, have been conducted [39].

Food products found on the market that use natural dyes.

Some food industries use dyes obtained from natural sources. This can generate opportunities for the development of food products with positive economic and environmental impacts [92]. This trend can be seen in some products in the supermarket, such as dry pasta that is naturally colored with dry tomatoes and spinaches. However, in Brazil, lycopene from tomatoes is still minimally used as a dye, except in some dry pasta, powdered foods, and some snacks [93].

Natural dyes, such as annatto, turmeric, mealybug carmine, and their combination, are more common in several products from dairy to chocolate. Generally, strawberry flavored cookies have carmine in their composition, which is a natural coloring extracted from cochineal (insect) and/or annatto coloring. In most churros and chocolate-filled cookies, the presence of the natural annatto or cochineal carmine has been observed to intensify the brown color; however, the caramel IV coloring is also part of the composition [94].

In the dairy group, most products flavored with strawberries are also added with natural dyes, such as mealybug carmine and annatto; however, some of them have also been added with synthetic dye. In fruit-flavored dairy products as well as yellow and red fruits, there is presence of annatto dye, mealybug carmine, and β-carotene.

Some wholegrain snacks, ready-made pasta for whole grain breads, and cassava flours use turmeric to add yellow color. Some chocolate bars filled with passion fruit have turmeric and annatto dyes. Some food supplements brands use the red color in beets to add color to certain products. Some industrialized products with natural dyes are shown in Table 3.

### 2.4. How Has Tomato Been Used in Food?

#### Use of Tomatoes as Coloring and Adjuvants in Food

In this study, only 14 articles were found on the use of tomatoes as food additives. This reveals an opportunity for future studies in this area, considering that tomatoes have numerous nutritional and technological advantages. Most of the articles investigated the use of tomatoes as nutritional additives with preservative and antioxidant functions but with less importance on coloring function.

Bakery and meat products are the most investigated foods with the addition of tomatoes, either by coloring or biological power (Table 4). In studies on macaron and sausage production, the color was highlighted with the addition of powdered tomatoes. There was an increase in the aesthetic function in macarons due to the color presented. In sausages, in addition to enhancing the color, the presence of the tomatoes led to an increase in the antioxidant activity [107,108]. Due to the coloring power of tomatoes, in addition to its application in food (Table 4), the use of tomato as coloring has the potential to be used in beverages.

Tomatoes have been added to food for different purposes (Table 4). Of the 14 articles evaluated, 11 showed color analysis. All articles presented sensory analysis with trained or untrained judges, two articles evaluated the carotenoid content, and seven articles evaluated the lycopene content, antioxidant activity, and pH. In addition, some of the articles also conducted shelf-life analysis.

Regarding the color analysis, in all 11 articles, the values of L* (luminosity) tend to decline with increase in the concentration of tomato by-products. In a study with sausages added with powdered tomato peels, the L* value decreased over the days [114]. In this study, a shelf-life test was performed over a period of 36 days.

Doménech-Asensi et al. [109] developed mortadella with the addition of tomato extract. The shelf-life analysis was within a duration of two months, with determinations on day 0, 30, and 60. In this case, the L* value decreased on day 30 and increased on day 60. However, the values of this variation were not expressive.

In the study in which light pork sausage was produced with the addition of powdered tomatoes [107], analysis of shelf life was also performed within the period of 30 days. Sausages with 1.5% tomato powder were unstable during storage in terms of color parameters, although no differences were observed with 0.8% tomato powder (control) and 1.2% tomato powder.

Regarding the values of a* (red/green) and b* (yellow/blue), an increasing trend was observed according to the addition of tomato by-products in all studies, such that b* values ranged from 6.25 ± 0.25 to 46.57 ± 0.82 and a* values ranged from −1.68 ± 2.26 to 37.7 ± 0.04. However, products that underwent cooking, such as hamburgers and spaghetti, recorded a decrease in a* values and a slight increase in b* values. In beef burger, the values of a* dropped by an average of 17 points after cooking, whereas in the sample with 3% tomato skin, the value of a* dropped from 33.88 ± 2.83 to 13.37 ± 1.67, while for the sample with 6% tomato skin, the b* value dropped from 17.14 ± 2.53 to 17.84 ± 1.62 [8].

In spaghetti, the value of b* increased by an average of 8 points, whereas in the sample with 15% lyophilized tomato, the value of b* increased from 33.50 ± 0.48 to 46.57 ± 0.82 after cooking and a* reduced by an average of 11 points. In the sample with 5% tomato, the value of a* dropped from 23.96 ± 0.78 to 11.86 ± 0.41 and L* decreased approximately by 2 points after cooking. Furthermore, regarding the sample with 5% tomato, the value of L* reduced from 70.07 ± 1.04 to 66.50 ± 0.78 [112]. In these studies, raw and cooked products were compared using color analysis.

In studies that produced other products that were cooked without comparison with raw food, such as macarons [108], cookies [115], crackers [116], muffins [117], and bread [118,119], the values of b* (44.76 ± 1.22) were higher than those of a* (23.28 ± 1.12), as observed in the bread sample with 35% tomato by-product [117]. This behavior can be explained by the change in the chemical structure of the carotenoids, since they undergo isomerization from *trans* to *cis* form at high temperature, with loss of color and increased bioavailability [25].

Regarding the analysis of carotenoids, only three studies reported this assessment. These studies evaluated the carotenoid content in products, such as spaghetti [112], hamburger [8], and bread [119]. In the case of spaghetti, with the addition of lyophilized tomato, a decay of total carotenoids in the sample with 5% tomato was noted, with values ranging from 55.59 ± 0.69 to 49.33 ± 0.88 µm/g. In the 10% tomato sample, the value ranged from 95.96 ± 2.29 to 83.74 ± 2.11 µm/g, and in the 15% tomato sample, the value ranged from 130.66 ± 1.10 to 117.84 ± 1.57 µm/g, with an average loss of less than 14% when compared to raw pasta due to cooking by boiling, which causes loss of solids [112].

In bread, carotenoid analysis was used to determine the composition of tomato, peel, and seed by-products, and a concentration of 174 mg/kg lycopene in the by-products was recorded [118]. In the study that produced beef hamburger, the level found in the sample with 4.5% tomato by-product was 4.9 mg/100 g, which is close to the recommended daily intake of lycopene of about 4 to 35 mg/day [29]. In general, the studies that evaluated the lycopene content observed a reduction (due to heat isomerization) when the product was subjected to cooking following the solvent method.

ANVISA included lycopene in its claims of approved functional property with the following statement: “Lycopene has an antioxidant action that protects cells against free radicals. Its consumption must be associated with a balanced diet and healthy lifestyle [73].

The American Dietetic Association establishes that the consumption of half cup a day = 30 mg or 10 servings/week (1 serving = half cup of tomato sauce) of tomatoes reduces the risk of prostate cancer [120].

Considering the antimicrobial activity of tomatoes, some products, such as sausage [108,114], mortadella [109], pork hamburger [110], and hamburger [8], were free from microbial contamination at the time of storage. Some authors suggest the reduction of nitrite. Even in the isolated case of the hamburger, it was concluded that there would be no need for the use of synthetic preservatives, since the addition of tomato extract increased the shelf life and limited microbial growth [8].

Regarding antioxidant activity, for mortadella, it ranged from 0.75 to 1.00 mm eq. Trolox/kg [109]. In spaghetti, it was possible to observe a slight increase in antioxidant activity after cooking, since the sample with 5% lyophilized tomato exhibited 71.32% antioxidant activity, which increased to 72.5% after cooking [112]. In this study, the method for obtaining the antioxidant activity was the sequestration of the radical 2,2-azinobis (3-ethylbenzothiazoline-6-sulfonic acid) (ABTS), which is considered the most adopted measure of the total antioxidant activity in compounds of lipophilic and hydrophilic nature, including flavonoids, carotenoids, and plasma antioxidants [121]. In other studies, the 2,2-diphenyl-1-picryl-hydrazil (DPPH) radical capture method, was developed to determine the antioxidant activity of various substances [122], was also used.

In the study involving the development of cheese, it was observed that the antioxidant activity over three months gradually declined until the 60th day; as analyzed in the sample with 1% tomato powder, it was 37% on the 1st day, 18% on the 20th day, 16% on the 40th day, and 14% on the 60th day, with a slight increase on the 90th day to 17% [113].

Generally, results of the sensory analysis showed good acceptance. Studies involving the production of sausage [107,114], macaron [108], pork burgers [110], nuggets [111], and cookies [115] were evaluated by trained judges, whereas the studies that produced mortadella [109], spaghetti [112], cheese [113], beef burgers [8], crackers [116], muffins [117], and bread [118,119] were evaluated by untrained judges. Among the studies evaluated, 12 presented sensory evaluation using a structured hedonic scale and two presented sensory evaluation using an unstructured linear scale.

The global impression is understood by the group relating to the first impression caused by the product as a whole, without representing the average scores of the other evaluated characteristics [123]. The values obtained for this parameter were in the range of 4.4–8 points for pork sausage [107], spaghetti [112], beef burgers [8], muffins [117], and bread [118,119], since these studies used a 9-point hedonic scale. In the articles that used tomato in macaron [108] and crackers [116], with a hedonic scale of 7 points, the values obtained were in the range of 4.5–5 points. In surveys that used a hedonic scale with 10 points, in which bologna [109] and sausage [114] were produced, there was a variation from 7 to 9 points. For evaluation of tomato-embedded cheese, a 5-point hedonic scale was used to analyze the sensory attributes [113], with the global impression varying from 4 to 5 points and, when using an 8-point hedonic scale that incorporated tomato powder into nuggets [111], these values were in the range of 6.7–7.2 points.

According to Teixeira et al. [124] and Dutcosky [125], for a product to be acceptable in terms of sensory properties, it is necessary to obtain an Acceptability Index (AI) of at least 70% in all attributes. Based on the grades for acceptability and AI calculation, the studies that produced pork sausage [107], macarons [108], pork burgers [110], nuggets [111], cheese [113], and crackers [116] showed good acceptability with formulations of powdered tomato by-product of 4%, 2%, 20%, 2%, 0.8%, and 5%, respectively, which presented AI greater than 70% for all attributes. As a common point, all the analyses had good attributes in terms of the items’ color, flavor, smell, and general acceptability. In the studies that produced macarons [108] and cheese [113], the greatest acceptance was in the versions with the lowest number of powdered tomatoes. In the production of mortadella, the best acceptance was recorded at the concentration of 10% [109].

Based on these articles, there are many gaps to be filled. Technological improvements are required before using tomatoes as a colorant in foods. These technological improvements are especially needed to reduce isomerization from *trans* to *cis* form, which despite optimizing bioavailability affects the antioxidant activity and color of the food.

## 3. Conclusions

The addition of tomatoes as a coloring and adjuvant in foods has many gaps and has created the opportunity for future researchers. In general, the addition of red tomato products in foods can reduce the oxidative process and improve the shelf life of foods. Tomatoes are sources of lycopene, and the addition of tomato skin powder is excellent for preserving hamburgers. In cooked foods, isomerization from *trans* to *cis* form can cause color loss but an increase in lycopene bioavailability. In the food industry, the use of tomatoes as a food coloring agent is limited to dried pasta and products that use red tomato products in their composition and generally improve sensory acceptance. However, the use of red tomato products as a food colorant is still a challenge, given the thermal sensitivity of the bioactive compound, which causes color instability. An alternative way to protect carotenoids from isomerization and/or oxidation would be to encapsulate the lycopene extracted from tomatoes.

## Figures and Tables

**Table 1:** Scientific reports on the toxicity of synthetic dyes.

Dyes	Toxicity	Source
Tartrazine	Carcinogenic activity/potential genotoxicity	[41,42]
Yellow twilight	Mutagenic activity	[43]
Amaranth	Carcinogenic and mutagenic activity, cytotoxicity, clastogenic and potential cytostaticity/genotoxicity	[42,44,45]
Erythrosine	Mutagenic activity, cytostaticity, cytotoxicity/potential genotoxicity	[42,46]
Patent blue V	Allergic activity, ability to alter the secondary structure of hemoglobin	[47,48]
Blue indigotina	Mutagenic activity	[49]
Red 40	Associated with kidney, stomach, lung and colon diseases, and anemia	[50]

Source: adapted from Zanoni and Yamanaka [39].

**Table 2:** Foods in which the use of dyes is not authorized.

Food	Legislation
Sugar	CNS/MS Resolution No. 04/1988 [71]
Sweetener	SVS/MS Ordinance No. 39/1998 [72]
Cereal-based food for infant feeding	Resolution RDC No. 27/2004 of ANVISA/MS [73]
Transitional foods for infants and young children	Ordinance No. 35/1998 of the SVS/MS [74]
Rectified alcoholic beverages (except Geneva) Arac, Mistela, Sangria	Resolution RDC No. 5/2013 of ANVISA/MS [75]
Fresh and frozen meats and raw salted products	Ordinance No. 1004/1998 of SVS/MS [76] and Normative Instruction No. 51/2006 of MAPA [77]
Processed cereals, wheat flour, and packaged wheat flour (wheat flour with added additives), premixes based on wheat flour (wheat flour with added additives and other ingredients for specific uses), other cereal flours, pastry dough and the like, pizza dough, pasta with vegetables	Resolution RDC No. 60/2007 of ANVISA/MS [78]

Source: adapted from Martins [69].

**Table 3:** Industrialized products with natural dyes according to manufacturers’ website.

Natural Dye	Industrialized Product [Source]
Natural dye carmine	Strawberry pulp yogurt [95]
Natural cochineal carmine and synthetic ponceau red 4R dye	Strawberry bilayer milk drink [95]
Annatto natural dye	Passion fruit liquid yogurt [95]
Natural dye carmine	Fermented milk drink strawberry [96]
Annatto natural dye	Fermented milk drink with yellow fruit pulp [96]
Natural dyes carmine and annatto	Fruit vitamin milk drink [97]
Natural dye carmine	Biscuit stuffed strawberry [98]
Carmine natural coloring and caramel coloring IV	Biscuit strawberry ice cream [98]
Carmine natural coloring and caramel coloring IV	Biscuit stuffed churros [98]
Natural dye carmine	Strawberry yogurt [98]
Carmine natural coloring and caramel coloring IV	Fermented milk strawberry [98]
Natural dyes carmine and annatto	Fruit smoothie yogurt [98]
Natural dyes β-carotene and annatto	Yogurt passion fruit, oats and ginger [98]
Dehydrated tomato and spinach	Pasta Tricolor Penne [99]
Dehydrated tomato and spinach	Pasta Grano Duro Tricolori Penne [100]
Natural dye carmine	Strawberry stuffed cookie [100]
Natural dye carmine	Biscuit strawberry [100]
Dehydrated tomato, dehydrated spinach, mealybug carmine, turmeric, and annatto	Pasta semolina mushroom tricolor [101]
Dehydrated tomato and spinach	Penne tricolore [102]
Natural turmeric dye	Corn chips [103]
Natural turmeric dye	Onions chips [103]
Natural turmeric dye	Ready dough for *mandioquinha* bread [103]
Natural turmeric dye	Ready dough for sweet potato bread [103]
Natural beet red dye	Arginine Power—Watermelon [104]
Natural curcumin dyes, mealybug carmine, beta-carotene, anthocyanins, Spirulina algae concentrate	Yogurt with Fragola frullata [105]
Concentrated natural beet and grape dyes	Creamy Yogurt Ciliegia in Pezzi [105]
Natural turmeric dye	Yellow cassava flour [106]
Natural dye turmeric and annatto	Chocolate bar [106]
Natural turmeric dye	Potato chips, Ripz Barbeque [106]
Dehydrated tomato and spinach	Tricolor Fusilli Pasta [106]
Dehydrated tomato and spinach	Prince Tricolor Rotini [106]
Spinach and tomato powder	ZPasta Tri Color Penne—Bronze Cut Artisan Pasta [106]

**Table 4:** Studies of tomatoes for the development of new products.

Objective	Food Developed	Ingredients and Proportions	Main Results and Conclusions	Source
To evaluate the effectiveness of different levels of tomato powder in inhibiting lipid oxidation and microbial growth, affecting color stability, as well as improving the sensory characteristics of sausages with low fat and salt content during refrigerated storage	Light pork sausage (stability test performed at 4 °C for 30 days, with measurements on days 0, 15, and 30)	Lean pork, fat substitutes, olive oil, NaCl:NaNO2-991 (salting), phosphate, sugar, monosodium glutamate, spices, ice, and powdered tomatoes. Proportions of tomato powder: 0 (control), 0.8%, 1.2%, and 1.5%	**Color parameters:** Sausages with 1.2 and 1.5% tomato powder had lower values (*p* < 0.05) for L* but values for a* and b* were higher (*p* < 0.05) than sausages without tomato powder during storage. The changes observed in the color parameters were mainly related to the added concentration of tomato powder; **Sensory analysis:** There were no differences in the color, aroma, flavor, tenderness, and juiciness values throughout the storage period; however, flavor scores were higher for sausages added with tomato powder at all storage stages; **Conclusions:** The low-fat pork sausage with added tomato powder showed lower (*p* < 0.05) lipid oxidation, L*, pH, and hardness values and higher a*, b*, and water-holding capacity values compared to control during refrigerated storage.	[108]
To develop macarons added with tomato powder	Macaron	Egg whites, sugar, powdered sugar, almond flour, and powdered tomatoes (0%, 5%, 10%, and 15%)	**Color parameters** (no stability test was performed): Considerable variation was observed with any increase in the concentration of powdered tomatoes. The L* value decreased with increasing concentration, with a darkening effect. a* and b* values increased with intensification of color; **Sensory analysis:** Regarding color and general acceptability, samples with 5 g and 10 g had higher scores, while samples with 15 g had lower scores than control; **Conclusions:** The L* value decreased with the increase in powdered tomatoes, whereas the factors “a” (red) and “b” (yellow) increased with the addition of powdered tomatoes. As the proportion of tomato powder increased, the texture of the macaron became softer. With the sensory analysis, it was possible to conclude that the macaron added 5% tomato powder had greater acceptance.	[109]
To evaluate the effect of adding tomato paste in mortadella in order to improve quality, increase nutritional properties, and reduce the lipid oxidation process associated with lycopene content	Mortadella	Group 1: raw meat, water, salt, soy protein, potato starch, skim milk, spices, dextrose, sodium polyphosphate, monosodium glutamate, trisodium citrate, sodium ascorbate and sodium nitrate. Addition of tomato paste in proportions of 2%, 6%, and 10%;Group 2: salt, dextrose, soy protein, corn dextrin spices, sodium polyphosphate, monosodium glutamate, trisodium citrate, sodium ascorbate, sodium nitrate and mealybug two regular mortadella, one with the addition of 10% tomato paste	**Color parameters** (stability test was performed at 4 °C for 60 days, with measurements on day 0, 30, and 60): There was a slight variation in the stability test, which was considered insignificant. The parameter b* had the most expressive variation, which can be associated with a slight fading; **Lycopene:** In group 1, it increased, varying between 0.21 and 1.99 mg/100 g, based on the percentage of tomato extract incorporated. In group 2, it was not detected in regular mortadella, since when there was 10% tomato extract, 1.65 mg/100 g lycopene was detected. During the storage period of 2 months, in the 2% and 6% samples, the concentration of lycopene was quite constant, with little change during storage; **Antioxidant activity by ABTS (mm eq. Trolox/kg):** Antioxidant activity was in the range of 0.75–1.00; **Sensory analysis:** Aspects, such as aroma, flavor, and general acceptability, showed an acceptability above 60% in mortadella added with tomato extract; **Conclusions:** Addition of the tomato extract resulted in a product with similar technological characteristics and improved nutritional and functional properties without affecting the sensory attributes of color, flavor, texture, or general acceptance. Moreover, the addition of tomato paste improved the stability of the mortadella during the useful life period, thereby reducing the lipid oxidation associated with storage.	[110]
To evaluate the effect of incorporating tomato paste at different levels under high pressure treatment in pork burgers to develop new healthier meat products with extended shelf life	Pork burger	Meat, tomato paste, wheat flour, salt, parsley, garlic, onion, black, and white pepper. Proportions of tomato paste: 0%, 5%, 10%, 15%, and 20%	**Color parameters** (stability test was performed at 4 ± 1°C for 30 days, with measurements on day 0, 15, and 30): There was a slight variation in the stability test, which was considered insignificant. Since the parameter b* is the most expressive, which can be associated there is a slight fading; **Lycopene:** By comparing the samples with and without discharge processing, it was possible to observe that there was a minimal difference between samples with lower proportion of tomato paste, which was considered insignificant. In the sample with 20% tomato paste, there was a significant difference; **Sensory analysis:** In the color parameter, it was observed that the higher the proportion of tomato paste, the greater the acceptance of the evaluators. There were differences regarding samples with and without high pressure processing, which was considered insignificant; **Conclusions:** The tomatoes paste increased the shelf life during storage and limited the growth of microorganisms. Hamburgers with a greater amount of pasta showed greater stability to lipid oxidation during storage. Therefore, it is not necessary to increase production with synthetic preservative additives. There were color differences while the hamburgers were still raw, whereas after cooking, this variation was no longer noticeable.	[111]
To evaluate the effect of different levels of pomegranate seed powder and tomato powder on the quality characteristics of chicken nuggets	Chicken nugget	Chicken, soy oil, water, spice mix, wheat flour, spice mix, salt, sucrose, sodium triphosphate, and sodium nitrite. Different levels of powdered tomato and powdered pomegranate seed;Group 1: tomato powder: 0%, 1%, 2%, and 3%;Group 2: pomegranate seed powder: 0%, 1%, 2%, and 3%	**Sensory analysis:** In terms of color parameter and general acceptability, they obtained a higher score than control.	[112]
To analyze the effects of incorporating unconventional vegetable ingredients on the development of pasta, such as freeze-dried tomatoes	Spaghetti pasta	Freeze-dried tomato, special wheat flour, powdered egg and water, durum wheat flour (100%, 95%, 90%, and 85%) and freeze-dried tomato (0%, 5%, 10%, and 15%)	**Color parameters** (no stability test was performed): There was a slight variation when comparing the raw and cooked food. The values of L* and a* declined. The b* parameter was the most expressive, since there was a slight increase in this value in samples with more than 10% freeze-dried tomato after cooking; **Lycopene:** The addition of lyophilized tomato added lycopene to the product. There was a loss in the concentration of this carotenoid with cooking; **Antioxidant activity:** With the addition of lyophilized tomato, there was an increase in antioxidant activity. This activity also increased slightly after cooking; **Sensory analysis:** A higher score (score above 6) was obtained in the color parameter and general acceptability when compared to control; **Conclusions:** The addition of lyophilized tomato in fresh spaghetti caused significant changes in the technological quality of the samples. Despite this, based on the comparison with other studies, it can be inferred that the products developed showed acceptable quality characteristics in cooking.	[113]
To improve the functional and nutritional attributes of processed cheese and to investigate the rheological, physical-chemical, and sensory characteristics of processed cheeses containing tomato powder	Cheese	Feta cheese, water, butter, emulsifying salts, and powdered tomatoes. Proportions of tomato powder: 0% (control), 1%, 2%, and 4%	**Lycopene:** In the stability test, decay was observed in all samples except in the control sample, since there was no concentration of lycopene; **Sensory analysis:** It was possible to observe that with regard to color, the samples had good acceptance, though with insignificant difference, since it was above the control sample (i.e., approaching 5 points). Regarding general acceptability, cheeses with the addition of powdered tomatoes stood out considerably above the control sample (i.e., approaching 5 points); **Conclusions:** The processed cheese samples containing the powdered tomato did not present different values of protein, fat, and moisture content. It presented higher levels of lycopene, which is an interesting finding, judging from a nutritional point of view. The sample with the highest acceptance was the one that contained 2% tomato powder.	[114]
To investigate the microstructural character and the binding capacity of sausages with tomato peel powder treated by different processes and resulting in different particle diameters	Sausage	Beef and pork, pork fat, pre-emulsified fat and tomato powder produced by two methods (conventional mode and one with air flow) six versions: high fat content, low fat content, and one of each with the addition of the powdered tomato peel from each method	**Color parameters** (stability test was done at 4 °C for 48 days, with measurements on day 0, 24, 36, and 48; however, the color parameter measurements for day 48 were not included): The value of L* declined with the stability test in all samples, in the sample with the smallest proportion, a slight increase in parameter a* was observed, while parameter b* varied increasingly until day 24 and slightly decreased on day 36, thus highlighting the sample with a higher proportion of tomato powder by-product, as it slightly increased; **Sensory analysis:** It was possible to observe that with respect to color, the control samples received higher scores between 8 and 9, which was followed by sausage with low fat content. The addition of tomato powder by the conventional method scored just above 8. With respect to the general acceptability, the control sausages had a higher score between 8 and 9. The sausage with a higher fat content scored closer to 9; **Conclusions:** With the addition of tomato skin flour, it was possible to observe that there is an improvement in the texture of the low-fat sausages. Sausages with a low dose of tomato peel had greater public acceptance.	[115]
To determine the maximum concentration of dried tomato peel that can be added to hamburgers to obtain a product enriched in lycopene, with minimal changes in the physicochemical and sensory properties	Beef burger	Minced meat and tomato skin. Proportions: 1.5 g, 3 g, 4.5 g, and 6 g of tomato skin powder per 100 g of meat	**Color parameters** (no stability test was performed): There was a slight variation when compared to the raw and cooked food. The parameters L* and b* increased slightly with cooking, while a * declined after cooking; **Sensory analysis:** In relation to color, the control sample had a score above 6.8. Only the sample with 1.5 g of tomato by-product had a score above 6. Regarding general acceptability, scores between 4 and 6 below the control sample were obtained; **Conclusions:** The hamburgers have a characteristic reddish color as a result of the addition of the tomato by-product. It was evaluated that the sensory qualities remained acceptable until the concentration of 4.5%. The amount of lycopene contained in the product is close to the recommended daily intake of about 4.9 mg per 100 g of product.	[8]
To examine the influence of the addition of dried tomato pomace on the physical and sensory properties of whole rye flour crackers	Whole rye flour cookie	Integral rye flour, refined sugar, vegetable fat, sodium chloride, sodium bicarbonate, ammonium bicarbonate and water (added until it reaches 18% moisture in the dough). Tomato pomace was used to replace part of the whole 0%, 15%, and 25% rye flour	**Color parameters** (no stability test was performed): Considerable variation was observed with the increase in the concentration of powdered tomatoes. L* decreased slightly with increasing concentration, with a darkening effect. a* and b* increased with the slight intensification of color; **Sensory analysis:** Regarding color, the samples had good acceptance and, in equality with the control sample, scoring close to 100 points. Regarding general acceptability, cookies with added powdered tomatoes considerably stood out above the control sample; **Conclusions:** The level of incorporation of the tomato by-product altered several properties of the cookies, such as the spreading factor, hardness, and color of the cookies. This study demonstrated the potential of using the portions of tomato pomace investigated in the production of biscuits to obtain adequate textural and sensory properties of the final product. According to the results of the sensory analysis, the level of substitution of 15% caused a decrease in the roughness, fracture, and granularity of the surface, as well as an increase in the intensity of flavor. The 25% substitution level caused a greater tomato flavor and softening of the biscuit.	[116]
To determine the potential use of tomato paste residues that are generally used in animal feed and are rich in biologically active components in the human diet. The use of residues in the human diet can also reduce the problem of environmental pollution	Cracker	Wheat flour, dried tomato pomace, wheat starch, water, corn oil, sugar, salt, and chemical yeast. Proportion of tomato by-product: 0%, 4%, 8%, and 12%	**Color parameters** (no stability test was performed): Regarding color parameters, considerable variation was observed with the increase in the concentration of the powdered tomatoes. The value of L* decreased slightly with increasing concentration, with a darkening effect. a* and b* increased with a slight intensification of color; **Antioxidant activity:** They showed greater antioxidant activity than the control sample; **Sensory analysis:** Regarding color and general acceptability, only samples with 4% and 8% of the tomato by-product scored above the control sample; **Conclusions:** Addition of the tomato pomace powder increased the crude protein, soluble, insoluble, and total dietary fibers, minerals, total phenolic levels, and total antioxidant capacity of the crackers. Cookies with tomato pomace powder presented color values (a* and b*) that are higher than the control, and the colors of the samples with 4% and 8% had higher scores in the sensory analysis, although without difference with respect to the others. The panelists liked the cookies equally in terms of color, smell, flavor, crispness, and general acceptability. However, the sensory evaluation indicated that the wheat flour substitution greater than 12% with tomato pomace powder in the production of biscuits was not recommended.	[117]
To develop breads and muffins incorporated with tomato processing residues in order to promote a longer shelf life	Bread and muffin	Tomato pomace, wheat flour, sugar, salt, dry yeast, butter, baking powder, and powdered milk. Bread: 35% wheat flour (weight) in tomato by-product. Muffin: 40% wheat flour (weight) in tomato by-product	**Color parameters** (no stability test was performed): Regarding color parameters, considerable variation was observed with the increase in the concentration of the powdered tomatoes. L * decreased slightly with increasing concentration, with a darkening effect. a * and b * increased with a slight intensification of color; **Lycopene:** A considerable amount of lycopene in the samples;Antioxidant activity: An increase in antioxidant activity was observed; **Sensory analysis:** Both bread and muffin had a higher rating than the controls, scoring above 8 on both products, in terms of color and general acceptability, whereas the control scored below 8. Regarding the overall acceptability, the two products had good acceptance; however, they were slightly below of the control scores, scoring between 8 and 9, with the control sample scoring slightly above; **Conclusions:** Most widely accepted products contained 35% tomato pomace and improved several aspects of bread and muffins, such as dietary fiber, vitamin C, antioxidant activity, and minerals. It also brought acceptable properties of color and texture. An increase in the shelf life of products was observed.	[118]
To investigate the incorporation of tomato seeds in bread with the aim of improving the bread quality	Bread	Wheat flour, yeast, salt, tomato powder, and water. Proportion of tomato by-product: 0% (control), 5%, 10%, 15, and 20% flour (weight)	**Color parameters** (no stability test was performed): Regarding the color parameters, considerable variation was observed with increase in concentration of the powdered tomatoes. L* decreased slightly with increasing concentration, with a darkening effect. a* and b* increased with a slight intensification of color; **Sensory analysis:** Regarding color, all samples had good scores between 7 and 9, with insignificant differences. With respect to general acceptability, the control bread with 5% and 10% tomatoes had the highest score (between 7 and 9); **Conclusions:** The technological and nutritional qualities of bread was improved. The color of the bread crumb was changed, making it more reddish. The ideal proportion was up to 10% tomato seed flour.	[119]
To evaluate a sample of tomato residue from the industry and consequently experiment with the addition of this residue to improve the technological qualities of bread	Bread	Refined wheat flour, salt, yeast, improver, water at 25 °C, and tomato powder. Proportion of tomato by-product: 6% and 10% flour (weight)	**Lycopene and antioxidant activity:** Only the concentration of the by-product used was analyzed and not the final product;Sensory analysis: All samples had a lower score than the control sample. The control samples had a score between 8 and 9, while the test samples with added by-product had a score between 7 and 8; **Conclusions:** Tomato residue is an excellent source of lycopene, and its incorporation in the bread recipe is a great solution for the use of the by-product. Bread supplemented with 6% tomato residue flour had the greatest acceptance.	[120]
